# Molecular evolution and adaptations of *Legionella pneumophila* from amoebae hosts to macrophages

**DOI:** 10.3389/fcimb.2026.1787137

**Published:** 2026-02-19

**Authors:** Cheon Jee Shin, Yousef Abu Kwaik

**Affiliations:** 1 Department of Microbiology and Immunology, University of Louisville, Louisville, KY, United States; 2 Center for Predictive Medicine, University of Louisville, Louisville, KY, United States

**Keywords:** bacterial pathogenesis, effector, evolution, genomics, host-pathogen interactions, protozoa, type II secretion system (T2SS), type IV secretion system (T4SS)

## Abstract

*Legionella pneumophila* is an environmental bacterium that emerged from a prolonged co-evolution and adaptation to free-living amoebae as the natural hosts. Within these protozoan hosts, *L. pneumophila* evolved to evade amoebae predation and remodel their vacuoles into endoplasmic reticulum (ER)-derived vacuoles that evade phagosomal-lysosomal fusion. The *L. pneumophila*-amoebae co-evolution fortuitously has facilitated infection of human alveolar macrophages, resulting in pneumonia known as Legionnaires’ Disease. Intracellular replication and host manipulation are orchestrated by the Dot/Icm Type IV Secretion System (T4SS), which translocate more than 350 effectors that remodel host membrane trafficking, metabolism, and immune signaling, and by the Type II Secretion System, which releases ≈120 hydrolytic enzymes that promote nutrient acquisition and environmental persistence. The extraordinary diversity and redundancy of these effectors reflect evolutionary pressures within diverse protozoan reservoirs that have sculpted an arsenal capable of subverting numerous eukaryotic processes in diverse environmental hosts and is obvious from genomic plasticity. Adaptation of *L. pneumophila* to the intracellular life within unicellular phagocytic amoebae has played a major role in host expansion to human macrophages that share numerous conserved processes with amoebae, which are thought to be their ancestors. However, since *Legionella* modulate various mammalian-specific processes not present in unicellular amoebae, it is also likely that *Legionella* has also evolved through interaction with multi-cellular eukaryotic environmental hosts prior the infection of humans. It is also possible that many of the mammalian-specific processes modulated by effectors of *Legionella* can be an accidental host response to amoebae-adapted effectors rather than specific adaptation. This is a comprehensive review that synthesizes advances in our knowledge of ecology, epidemiology, metabolism, secretion systems, and host-pathogen interactions of *L. pneumophila*, highlighting how environmental selection and co-evolution with protozoan hosts drive genomic evolution and expansion of the host range from unicellular eukaryotic amoebae to humans.

## 1 Introduction

### 1.1 History of Legionnaires’ disease

The first publicized outbreak of *Legionella* was in summer of 1976 at the annual American Legion Convention that was held at the Bellevue-Stratford hotel in Philadelphia, Pennsylvania (Fraser et al., 1977). There was a large outbreak of a mysterious pulmonary disease soon after the convention, and at least 182 attendees suffered from pneumonia-like symptoms and 29 of them succumbed to disease. In addition to the convention attendees, several pedestrians and bus drivers that passed by the hotel’s frontage also fell ill, resulting in a total of 221 patients that contracted the disease with 34 deaths (Fraser et al., 1977; McDade et al., 1977; Honigsbaum, 2016). The causative agent was unknown as the disease symptoms were not caused by any known microorganisms or toxins at the time. Bacteriologists, virologists, parasitologists, epidemiologists, and toxicologists were recruited at the Centers for Disease Control and Prevention (CDC) to collaborate and identify the responsible pathogen (McDade et al., 1977; Fraser, 2005).

The causative agent was finally identified by examining liver sections of guinea pigs that were inoculated with lung tissue samples from patients. It took six months from the outbreak to identify the pathogen due to its fastidious nutrient requirements for culture and growth (McDade et al., 1977). In addition, isolation of the bacterium was also difficult as it only replicated within alveolar macrophages of human and guinea pigs, making it difficult to isolate viable and culturable bacteria from host lung tissue samples or study in simpler animal models like mouse (McDade et al., 1977). In 1979, the rod-shaped bacteria that caused the mysterious pulmonary disease were given the name *Legionella pneumophila* due to the severe pneumonia outbreak it caused that affected many of the attendees of the American Legion Convention and the associated respiratory illness, Legionnaire’ disease (Brenner et al., 1979; Fraser et al., 1977; McDade et al., 1977). The source of *L. pneumophila* from the first publicized outbreak was unknown at the time as environmental testing could not identify the source of transmission, but epidemiological studies suggest that the air conditioning and cooling tower at the Bellevue-Stratfor Hotel were contaminated with the bacteria (Muder et al., 1986).

*Legionella pneumophila* are Gram-negative facultative intracellular bacterial pathogens belonging to the family Legionellaceae (Brenner et al., 1979). *L. pneumophila* is uni-flagellated bacilli that may adopt filamentous or cocci-like forms under certain growth conditions (Rodgers et al., 1980; Chandler et al., 1980; Pine et al., 1979; Katz et al., 1984; Piao et al., 2006). There are more than 60 *Legionella* species identified, and about 50% of these species are known to cause disease. *L. pneumophila* is responsible for approximately 90% of the Legionnaires’ Disease cases (Meier-Kolthoff et al., 2022; Muder and Yu, 2002; Newton et al., 2010). *L. pneumophila* can be subdivided into 16 serogroups based on antigenicity of the lipopolysaccharide (LPS) O-antigen (Khan et al., 2013). By far, serogroup 1 is most commonly associated with disease, causing more than 80% of laboratory confirmed cases of legionellosis (Yu et al., 2002; Palusinska-Szysz et al., 2019). Interestingly, *L. longbeachae*, which is present in the soil, is responsible for approximately 50% of the Legionnaires’ Disease cases in Australia and New Zealand (Ha et al., 2024).

### 1.2 Transmission

*L. pneumophila* is considered as an environmental bacteria that have co-evolved with amoebae hosts and seems to follow the prevailing dogma that the mode of transmission is from an environmental source to the dead-end human host (Baskerville et al., 1981; Zuravleff et al., 1983). There had only been one unusual, reported case of a person-to-person transmission of *L. pneumophila* (Correia et al., 2016; Borges et al., 2016). Studies have revealed that emergence of outbreaks of *L. pneumophila* in the last half of 20^th^ century is due to human alteration of the environment and generation of aerosols by new equipment such as cooling towers, air conditioning systems, pools, hot tubs, showers, misting machines, and humidifiers (Fields et al., 2002). Inhalation of contaminated aerosolized water droplets has been traced to human-made aquatic environments (Fields et al., 2002; Berendt et al., 1980; Davis et al., 1982; Hambleton et al., 1983; Muder et al., 1986). Left in the natural aquatic environment, *L. pneumophila* would be an extremely rare cause of human disease.

While *L. pneumophila* evolved within natural aquatic ecosystems, contemporary legionellosis is overwhelmingly linked to engineered water systems rather than pristine freshwater habitats. Plumbing networks, cooling towers, hot-water distribution systems, and healthcare water infrastructure generate warm, stagnant, biofilm-rich environments that promote amoebal proliferation and *L. pneumophila* amplification (Gleason and Cohn, 2022). Within these systems, biofilms concentrate nutrients and microbial biomass while limiting disinfectant penetration, thereby stabilizing niches in which amoeba-associated *L. pneumophila* can persist. Mechanistic modeling and field-based studies further demonstrate that hydraulic residence time, temperature stratification, pipe materials, and disinfectant decay collectively govern *L. pneumophila* persistence and outbreak risk in the engineered water environments (Ortiz et al., 2025). These observations underscore that modern *L. pneumophila* transmission is largely an emergent property of engineered ecological niches rather than a direct consequence of natural environmental exposure.

In addition to direct release of *L. pneumophila* following amoebal lysis or exocytosis, extracellular vesicles (EVs) have emerged as an underappreciated mechanism contributing to pathogen persistence and dissemination in protozoan-pathogen systems. Protozoa are capable of releasing membrane-bound vesicles containing host-derived lipids, proteins, and microbial cargo that can protect enclosed material from environmental stressors and facilitate intercellular communication (Alvarado-Ocampo et al., 2024). Notably, early experimental studies demonstrated that *Legionella-*infected amoebae can actively expel micrometer-scale membrane-bound vesicles containing viable *L. pneumophila*, which persist in aquatic environments and remain respirable, suggesting a direct role for amoebae-derived vesicles in environmental dissemination and transmission (Rowbotham, 1986; Berk et al., 1998; Bouyer et al., 2007) Although EV-mediated processes remain incompletely characterized for *L. pneumophila* at the molecular level, observations from other protozoan systems suggest that vesicle-associated bacteria or bacterial components can contribute to biofilm formation, environmental persistence, and delivery of virulence factors into new protozoan or mammalian host cells (Twu et al., 2013; Schwechheimer and Kuehn, 2015). By contrast, the contribution of host-derived EVs as a mechanism for *L. pneumophila* persistence, dissemination, or immune modulation during macrophage infection remains largely unexplored. Together, these findings support a model in which amoeba-derived vesicles, ranging from expelled bacteria-containing vesicles to smaller EV-like structures, may represent protective and communicative intermediates that bridge environmental survival, biofilm ecology, and intracellular pathogenesis in aquatic environments subject to fluctuating physical and chemical stressors.

### 1.3 Clinical manifestations

*L. pneumophila* infections result in two distinct clinical manifestations: Legionnaires’ Disease or Pontiac Fever (Fraser et al., 1977; Cunha et al., 2016; Fraser et al., 1979). Pontiac fever, named after the first publicized outbreak in Pontiac, Michigan, is a mild, flu-like illness that typically resolves within 5 days and does not progress to pneumonia (Fraser et al., 1979; Kaufmann et al., 1981; Burillo et al., 2017). Symptoms of Pontiac fever usually appear 5–72 hours after exposure (Tossa et al., 2006). Legionnaires’ Disease is the pneumonic form of legionellosis, with a fatality rate of ~10% in healthy individuals and more than 25% in high-risk patients (Burillo et al., 2017). Risk factors include elderly (age > 60), smoking, chronic illnesses, and immunosuppression (Doebbeling and Wenzel, 1987; England et al., 1981; Radaelli et al., 1991; Marston et al., 1994; Newton et al., 2010; Gump and Keegan, 1986).

Symptoms of Legionnaires’ Disease usually begin 2–10 days after exposure to environmental bacteria, with an average onset of 5–6 days (Egan et al., 2011). Symptoms include high fever, cough, headache, and fatigue (Fraser et al., 1977). In severe cases, Legionnaires’ Disease may present with loss of appetite, occasional diarrhea, renal failure, encephalopathy, and pericarditis (Cunha et al., 2016; Burillo et al., 2017). Patients who succumb to the disease usually die as a result of multi organ failure or respiratory shock (Marston et al., 1994). There are no vaccines currently available to protect against legionellosis, but infection can be treated with recommended classes of antibiotics including fluoroquinolones, tetracycline, and macrolides, while *L. pneumophila* is naturally resistant to penicillin and β-lactams (Pedro-Botet and Yu, 2006; Bopp et al., 2011; DumarcetAgence Francaise De Securite Sanitaire Des Produits DE, 2012).

Among the leading pathogens responsible for community-acquired pneumonia, Legionnaires’ pneumonia consistently ranks within the top three causes of pneumonia-associated hospitalizations (Graham et al., 2020; Bartlett, 2011). However, cases of pneumonia caused by Legionnaires’ Disease are likely underdiagnosed and under-reported in many countries due to lack of diagnostics and surveillance systems. Studies have suggested that cases of Legionnaires’ Disease that are reported to the CDC represent less than 5% of actual Legionnaires’ Disease patients (Burillo et al., 2017; Phin et al., 2014; Garrison et al., 2014). This is further supported by the data from the United States indicating that there was a 249% increase in estimated incidence of Legionnaires’ Disease from 2000–2011 due to continued efforts to actively report incidences (Dooling et al., 2015). Although sensitive diagnostic tools such as urine antigen ELISA and sequence-based assays are available to detect *Legionella* infections and determine strains, there is a lack of consistent patient testing and absence of standardized protocols for accurately estimating the annual incidence of confirmed Legionnaires’ Disease cases (Mangiafico et al., 1981; Mao, 1988; Leland and Kohler, 1991; Ratcliff, 2013).

From a public health perspective, *Legionella* represents a major cause of community-acquired pneumonia (CAP) responsible for at least 10-15% of cases. Global meta-analyses indicate that *Legionella* accounts for a substantial fraction of hospitalized CAP cases, particularly severe pneumonia. However, true incidence is likely highly underestimated due to widespread reliance on laboratory diagnosis using the urinary antigen testing (UAT), which primarily detects *L. pneumophila* serogroup 1 but fails to detect other serogroups strains or other pathogenic species of *Legionella* (Graham et al., 2022; Chambers et al., 2021). In addition, large number of CAP patients are treated with antibiotics without the laboratory diagnosis of the causative agent.

Recognition of engineered water systems as long-term selective environments has prompted growing interest in ecological control strategies that move beyond chemical disinfection alone. Probiotic and microbiome-informed approaches aimed at reshaping aquatic microbial communities to suppress *L. pneumophila* growth have shown promise in experimental and pilot-scale systems (Cavallaro et al., 2022). At the same time, chronic exposure to sublethal concentrations of disinfectants, metals, and other chemical stressors within plumbing systems is increasingly recognized as a driver of antimicrobial resistance development within biofilms and amoeba-associated bacteria (Hayward et al., 2025). These environments may therefore function as reservoirs of resistance traits with potential implications for clinical management of legionellosis. Accordingly, forward-looking frameworks emphasize integrated, ecology-informed interventions that incorporate microbial community dynamics, infrastructure design, and system maintenance rather than reliance on biocides alone (Hammes et al., 2025).

## 2 Environmental niche and ecology

One of the hallmark characteristics of *L. pneumophila* is that it proliferates within a wide range of unicellular eukaryotes as its natural aquatic hosts (Price et al., 2024; Best and Abu Kwaik, 2018). It has been shown that *Legionella* can replicate in at least 30 species of protozoan hosts across multiple phyla (Fields, 1996; Breiman et al., 1990; Fields et al., 1989, Fields et al., 1984; Hagele et al., 2000; Newsome et al., 1985; Rowbotham, 1980, Rowbotham, 1986; Smith-Somerville et al., 1991; Solomon et al., 2000; Tyndall and Domingue, 1982; Kikuhara et al., 1994; Tyson et al., 2014; Rasch et al., 2016; Scheikl et al., 2016; Price et al., 2024; Swart et al., 2018). These diverse interactions predominantly occur in aquatic and soil environments, where free-living protozoan hosts are commonly found (Wang et al., 2025; Shames, 2023). Early studies have laid the groundwork for establishing *L. pneumophila* as a model system for studying bacteria-protozoa interactions, yielding insights into host adaptation, intracellular parasitism, and pathogen evolution in natural reservoirs (Price et al., 2024). Many other bacterial pathogens have been shown to evade amoeba predation, including *Chlamydia*, *Coxiella*, *Rickettssia*, *Francisella*, *Mycobacteria*, *Salmonella*, *Bartonella*, *Rhodococcus*, *Pseudomonas*, *Vibrio*, *Helicobacter*, *Campylobacter*, and *Aliarcobacter*, and these have been recently reviewed (Price et al., 2024).

Protozoan hosts also play a crucial role in biofilm formation, further promoting *L. pneumophila* persistence in water systems. Interestingly, the density of *L. pneumophila* within biofilms correlates with protozoan biomass as several amoeba species are also associated with biofilm communities (Liu et al., 2012; Taylor et al., 2009). The structure or composition of the biofilm makes *L. pneumophila* containing biofilms extremely resistant to biocide treatments in man-made water systems (Emtiazi et al., 2004).

*L. pneumophila* found within biofilms or exposed to environmental stress may enter a viable but non-culturable (VBNC) state, which is characterized by low metabolic activity and resistance to standard culturing methods (Epalle et al., 2015; Dietersdorfer et al., 2018). The VBNC state has been shown to protect *L. pneumophila* from chlorination, and these VBNC forms can be resuscitated upon co-culture with protozoa, representing an additional survival mechanism under disinfection stress (Dietersdorfer et al., 2018; Epalle et al., 2015; Garcia et al., 2007).

The intracellular replication of *L. pneumophila* within free-living amoebae serves not only as a survival strategy in the environments but also as a critical biological training ground that enhances the pathogen’s virulence potential in mammalian hosts (Price et al., 2024). Studies comparing amoeba-grown and *in vitro* cultured *L. pneumophila* have confirmed that amoeba-grown bacteria to be more infectious and cause more severe disease in animal models (Barker et al., 1992, Barker et al., 1993, Barker et al., 1995; Abu Kwaik et al., 1997). In addition, studies have demonstrated that *L. pneumophila* grown within amoebae exhibits markedly greater infectivity in human macrophages compared to bacteria cultured in axenic media, with high motility, increased resistance to antibiotics, biocides, and disinfectants (Cirillo et al., 1994; Byrne and Swanson, 1998; Hammer and Swanson, 1999; Pruckler et al., 1995). These observations underscore the critical role of protozoan hosts not only in amplification of *L. pneumophila* but also in priming the pathogen for successful infection of evolutionarily distant mammalian cells.

**Figure 1:**
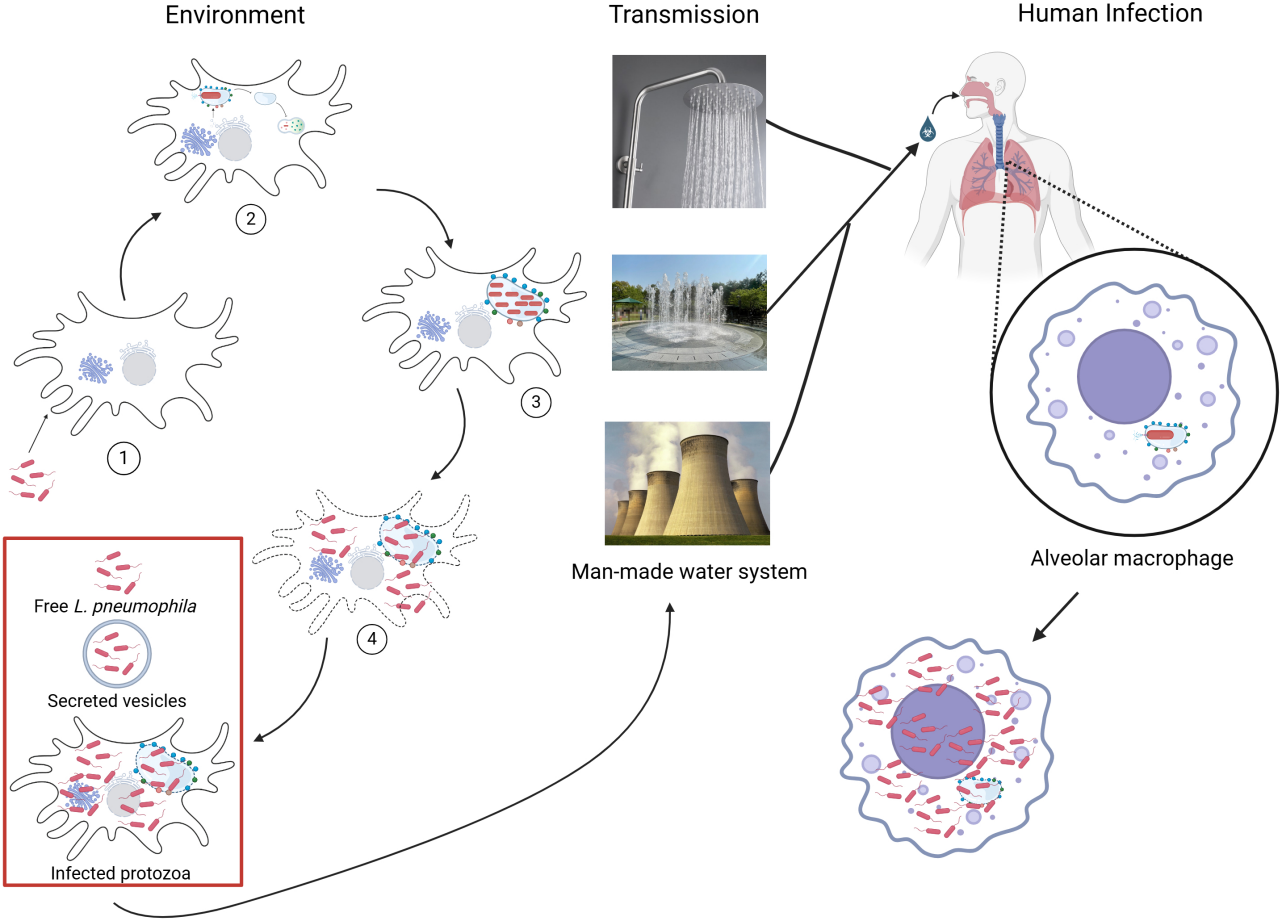
Environmental life cycle, transmission, and human infection of *L. pneumophila*. *L. pneumophila* alternates between intracellular replication within protozoa and a transmissive extracellular phase in aquatic environments. (1) Free *L. pneumophila* are phagocytosed by environmental protozoa such as *Acanthamoeba* or *Vermameoba* spp. (2) Inside the environmental hosts, *L. pneumophila* quickly intercepts the ER-derived vesicles and decorates its phagosome into a *Legionella-*containing vacuole (LCV) that evades endosomal-lysosomal degradation. (3) *L. pneumophila* successfully replicate within the LCV. (4) Upon nutrient depletion, *L. pneumophila* differentiate into the transmissive phase characterized by motility, stress resistance, and virulence factor expression and fill up the host cell cytosol. Upon host cell lysis or exocytosis, *L. pneumophila* is released into the environment as free bacteria, packaged in secreted vesicles from the host, or within infected protozoan hosts, enhancing survival in biofilms and water systems. Transmission to humans occurs via inhalation of contaminated aerosols generated from man-made water systems, including showers, fountains, and cooling towers. Once inhaled, *L. pneumophila* reach to alveoli of lungs, where they are engulfed by alveolar macrophages. In this new host, *L. pneumophila* establishes an LCV similar to that formed in protozoa, initiating intracellular replication.

Amoeba may serve as vectors for transmission. The *L. pneumophila*-infected amoebae can become aerosolized via water droplets or soil particles. Upon inhalation, these infected protozoa may facilitate delivery of *L. pneumophila* directly to the lower respiratory tract, where the bacteria can infect alveolar macrophages. This potential “Trojan horse” mechanism can enable *L. pneumophila* to bypass the initial host immune defenses, shielding the pathogen from both environmental insults and host surveillance systems, while simultaneously delivering it into a niche conducive to replication (Cianciotto, 2001; Price et al., 2024). This potential mode of bacterial transmission underscores the multifaceted importance of amoebae in *L. pneumophila*’s natural ecology and transmission dynamics (**Figure 1**).

Amoebae undergo differentiation between two developmental stages, a metabolically active trophozoite stage and a dormant cyst stage. In response to environmental stressors, including nutrient limitation, temperature shifts, osmotic stress, or chemical stress, amoebae undergo encystation, from the trophozoite into the cyst stage (Bouyer et al., 2007). Trophozoite stage of amoebae is permissive for *L. pneumophila* replication while the cyst stage restricts bacterial proliferation, but *L. pneumophila* remains viable for months within the cyst (Greub and Raoult, 2004; Ohno et al., 2008; Boamah et al., 2017; Abu Kwaik et al., 1998; Price et al., 2021; Kilvington and Price, 1990). Thus, encystation represents a survival mechanism for the protozoan host but restricts intracellular replication of *L. pneumophila*. For an intracellular pathogen like *L. pneumophila*, encystation poses a barrier to continued intracellular proliferation.

Several studies have demonstrated that *L. pneumophila* can persist within mature amoebal cyst for prolonged periods, up to six months or longer, without loss of viability (Abu Kwaik et al., 1998; Price et al., 2021; Ohno et al., 2008; Kilvington and Price, 1990). These bacteria remain metabolically quiescent and are protected from desiccation, heat, chlorine, and other common disinfectants (Ohno et al., 2008; Kilvington and Price, 1990; Richards et al., 2013). Once the cyst differentiates into a trophozoite stage, *L. pneumophila* resumes active replication, thus completing a cycle of persistence and proliferation that enables long-term environmental survival. Understanding these interactions at the molecular level may yield new targets for disrupting the persistence cycle of *L. pneumophila* in man-made aquatic environments. This ability to persist within cysts, resist disinfection, and subvert amoebal encystation not only explains the remarkable environmental resilience of *L. pneumophila* but also complicates public health efforts to eradicate the bacterium from engineered water systems.

Glycogen serves as a primary source for synthesis of the amoebal cyst cell wall, which is composed of cellulose or chitin (Byers et al., 1991; Lorenzo-Morales et al., 2008; Faber et al., 2017; Moon et al., 2014; Schaap and Schilde, 2018). A particularly striking example of interference with amoebal encystation by *L. pneumophila* involves the effector protein LamA, a Type IV secretion system (T4SS)-injected amylase that degrades host glycogen (Price et al., 2020). By abolishing glycogen stores in the amoeba host, LamA interferes with encystation, prolonging the window during which *L. pneumophila* can replicate within its amoebal host in the trophozoite stage (Price et al., 2020). Interestingly, when LamA is injected in human macrophages, it results a paradoxical pro-inflammatory response, which is an accidental macrophage response to an amoebae-adapted effector (Price et al., 2020).

### 2.1 Environmental complexity beyond protozoan hosts

Free-living amoebae that serve as natural hosts of *L. pneumophila* are rarely axenic organisms but instead represent complex intracellular ecosystems that frequently harbor additional bacterial symbionts acquired during grazing on bacteria (Rayamajhee et al., 2021; Greub and Raoult, 2004; Bozzaro and Eichinger, 2011; Price et al., 2024). Studies on *Acanthamoeba* have demonstrated that clinical isolates commonly contain *Chlamydia* spp., *Ricketsiales*, *Pseudomonas* spp., and *Mycobacterium* spp., whereas environmental isolates commonly contain *Legionella, Candidatus* spp., and other obligate or facultative intracellular bacteria (Rayamajhee et al., 2021). These co-resident microbes can influence host vacuolar trafficking, alter nutrient availability, and modulate cellular stress responses, thereby reshaping the intracellular niche encountered by *L. pneumophila* (Greub and Raoult, 2004; Rayamajhee et al., 2021; Thomas et al., 2010; Price et al., 2024; Schmitz-Esser et al., 2008). In addition, recent drinking-water microcosm studies provide compelling evidence that such microbial interactions can directly influence *Legionella* population dynamics, as illustrated by an apparent antagonistic relationship between *Neochlamydia* and *Legionella*, where the presence of one correlates with suppression of the other (Roman et al., 2025). Collectively, these observations indicate that the selective pressures shaping *L. pneumophila* evolution extend beyond amoebal predation alone and include competition with other intracellular microbes within protozoan hosts.

Beyond free-living protozoa, *Legionella* spp. are embedded within complex aquatic ecosystems that include multicellular eukaryotes, which commonly coexist with amoebae and diverse biofilm-associated microbial communities (Greub and Raoult, 2004; Fields et al., 2002). Although these multicellular organisms are not established hosts for *L. pneumophila*, their grazing, filter-feeding, and biofilm-associated lifestyles can directly or indirectly influence *Legionella* ecology and evolution by concentrating protozoa, organic matter, and microbial biomass within localized aquatic niches (Boamah et al., 2017; Declerck, 2010). Multicellular aquatic eukaryotes, such as snails, bivalves, and insect larvae, may act as ecological contexts and potential hosts that support environmental persistence of *Legionella* as direct or indirect hosts that have contributed to evolution of *Legionella* to infect humans (Fields et al., 2002). Consequently, while protozoa remain the well-established primary biological drivers of *L. pneumophila* evolution and intracellular adaptation, repeated exposure and possible adaptation to multi-cellular eukaryotic hosts may directly or indirectly reinforce pathogenic evolution of *Legionella*, and subsequent infection of human macrophages (Price et al., 2024; Shames, 2023). As discussed later in this review, ability of specific *Legionella* injected effectors to modulate various host processes of multi-cellular eukaryotes that are absent from unicellular eukaryotic hosts (such as NF-κB modulation by multiple specific effectors) may support the concept of adaptation of *Legionella* to multi-cellular eukaryotic environmental hosts prior to its infection of humans.

## 3 Molecular regulation of the bi-phasic lifecycle of *L. pneumophila*

*L. pneumophila* undergoes a tightly regulated biphasic life cycle that enables it to transition between two distinct physiological and morphological states: a replicative, non-motile phase and a transmissive, motile, and virulence phase (Byrne and Swanson, 1998). These phases are tightly controlled by environmental cues, particularly nutrient availability, and are central to the intracellular lifecycle and ecological persistence of *L. pneumophila* (Molofsky and Swanson, 2004; Bruggemann et al., 2006b; Faulkner and Garduno, 2002). During nutrient-rich conditions, *L. pneumophila* adopts a replicative form characterized by a non-flagellated, metabolically active, and non-cytotoxic phenotype (Molofsky and Swanson, 2004). Cells are long, rod-shaped, non-flagellated, and characterized by thin, wavy cell walls (Faulkner and Garduno, 2002). This stage is associated with exponential replication *in vitro* and within the *Legionella*-containing vacuole (LCV) (Newton et al., 2010; Isberg et al., 2009; Lifshitz et al., 2013; Hubber and Roy, 2010). The traits that are related to virulence and transmission are downregulated as those traits are not needed during this metabolically active replicative phase. During the exponential phase, the bacteria down-regulate transmission activators and up-regulate a repressor of transmission traits (Byrne and Swanson, 1998; Molofsky and Swanson, 2003).

Once the environment becomes nutrient-limited, particularly amino acid depletion, and disadvantageous for *L. pneumophila* replication, the bacterium initiates a tightly regulated developmental transition into the transmissive phase (Hammer and Swanson, 1999; Dalebroux et al., 2009; Sahr et al., 2017; Oliva et al., 2018). Interestingly, *L. pneumophila* undergo dramatic morphological changes including becoming shorter, thicker with smooth cell wall, and formation of inclusions of poly-3-hydroxybutyrate (PHB) (Faulkner and Garduno, 2002; Bruggemann et al., 2006b; Garduno et al., 2002).

The switch from replicative phase to transmissive phase at the post-exponential phase is mediated by the stringent response, triggered by the accumulation of guanosine 3, 5-bispyrophosphate (ppGpp) synthesized by synthetase enzyme RelA (Hammer and Swanson, 1999). In addition, fatty acid depletion leads to accumulation of ppGpp by SpoT (Dalebroux et al., 2009). Accumulation of ppGpp activates a transcriptional reprogramming cascade involving alternative sigma factors (RpoS and FliA), which in turn triggers the two-component regulatory system (LetA/LetS), and both RelA and Rpos are required for optimal intracellular replication of *L. pneumophila* (Hammer et al., 2002; Zusman et al., 2002; Bachman and Swanson, 2004; Abu-Zant et al., 2006). LetA activates non-coding RNAs (RsmY and RsmZ) that sequester CsrA, a post-transcriptional repressor of transmission traits, which induces expression of virulence genes, motility-associated proteins (flagella and pili), and transmission traits while repressing replication-associated genes (Bruggemann et al., 2006b; Edwards et al., 2010; Sahr et al., 2017; Tiaden et al., 2007; Schulz et al., 2012). This form, during the intracellular infection, can exit the host cell either through lysis mediated by cytolysins such as RtxA and MspA, or through non-lytic exocytosis mechanisms facilitated by effector proteins such as LepA and LepB (Chen et al., 2004; Eisenreich and Heuner, 2016). Once released, transmissive *L. pneumophila* can infect new hosts, persist as planktonic forms, or embed into biofilms (Garduno et al., 2002; Hilbi and Haas, 2012; Isberg et al., 2009; Robertson et al., 2014; Zhang et al., 2018). In response to extreme conditions, *L. pneumophila* may transition into a VBNC state or the mature infectious form (MIF), which is a highly differentiated, spore-like variant adapted for survival and infectivity in hostile environments (Garduno et al., 2002; Dietersdorfer et al., 2018; Epalle et al., 2015; Robertson et al., 2014). The life cycle is not merely a binary switch but a dynamic, highly coordinated process that reflects the bacterium’s adaptability to fluctuating environments.

## 4 Metabolic regulation in response to environmental cues

To proliferate within the confines of the LCV, *L. pneumophila* have evolved a highly flexible and adaptive metabolic strategy aligned with its invasion of diverse protozoan hosts and intracellular lifecycle. Early efforts to cultivate *L. pneumophila* in chemically defined media revealed that the bacterium relies primarily on amino acids, rather than sugars, for carbon and energy sources (Tesh et al., 1983; Eylert et al., 2010; Kunze et al., 2021; Häuslein et al., 2017; Pine et al., 1979; George et al., 1980; Ristroph et al., 1981). *L. pneumophila* can access and degrade host glycogen via a eukaryotic-like glucoamylase (GamA), expressed during intracellular replication (Bruggemann et al., 2006a; Herrmann et al., 2011). Notably, *L. pneumophila* does not generate ATP via classical glycolytic flux, but glucose metabolism occurs via the Entner-Doudoroff (ED) and pentose phosphate (PPP) pathways, supporting histidine and mannose biosynthesis (Eylert et al., 2010; Hauslein et al., 2016; Harada et al., 2010). Interestingly, despite lacking key canonical glycolytic enzymes, *L. pneumophila* retains gluconeogenic capacity (Keen and Hoffman, 1984; Eisenreich and Heuner, 2016; Gorke and Stulke, 2008). Energy production is mainly driven by the tricarboxylic acid (TCA) cycle, which functions as the central metabolic hub fueled by amino acid and fatty acid catabolism rather than glycolysis (Eylert et al., 2010; Eisenreich and Heuner, 2016; Häuslein et al., 2017). *L. pneumophila* preferentially metabolizes serine, cysteine, threonine, and branched-chain amino acids (Fonseca and Swanson, 2014; Hammer et al., 2002; Eylert et al., 2010; Gillmaier et al., 2016; Hauslein et al., 2016). Simultaneously, utilization of glucose is primarily channeled into anabolic pathways rather than energy production. Glycerol is similarly metabolized via gluconeogenesis and the PPP, with limited flux through the TCA cycle (Hauslein et al., 2016).

Among amino acids, serine and threonine were identified as key energy substrates, while cysteine was shown to be essential for growth, and ferric pyrophosphate had a stimulatory effect (Pine et al., 1979; George et al., 1980; Ristroph et al., 1981). This preference reflects adaptation to the host, which is rich in free amino acids but comparatively low in free sugars (Tesh et al., 1983; Eylert et al., 2010). This amino acid-centric metabolic program reflects both environmental adaptation and genetic constraint, as *L. pneumophila* is auxotrophic for many amino acids including arginine, cysteine, isoleucine, leucine, methionine, threonine, and valine, which is synchronous with amino acid auxotrophy of amoeba hosts such as *Acanthamoeba* (Pine et al., 1979; George et al., 1980; Ristroph et al., 1981; Price et al., 2014b). Transcriptomic and metabolic studies confirmed that *L. pneumophila* upregulate expression of genes consistent with aerobic metabolism and robust amino acid catabolism during exponential replication (Sauer et al., 2005; Bruggemann et al., 2006b).

Isotopologue labeling experiments revealed a bipartite metabolic model in which serine serves as the primary carbon, nitrogen, and energy source during exponential growth, entering the TCA cycle via conversion to pyruvate and subsequently to acetyl-CoA (Eylert et al., 2010; Gillmaier et al., 2016; Hauslein et al., 2016). Consistent with a non-glycolytic metabolic architecture, carbon flux is routed into the TCA cycle, reinforcing the TCA cycle as the primary engine of ATP generation and precursor biosynthesis during replication (Eylert et al., 2010; Oliva et al., 2018). Notably, more than 50% of labeled serine is incorporated into proteinogenic amino acids and PHB, a carbon storage polymer (Eylert et al., 2010). Isotopologue studies further confirm that glucose contributes to PHB and anabolic synthesis during intracellular growth, even though its addition in broth culture does not enhance growth rate (Tesh et al., 1983; Eylert et al., 2010). Saturated fatty acids, such as palmitate, are also used as carbon sources in the post-exponential phase and feed into energy production and PHB biosynthesis (Häuslein et al., 2017). As nutrient availability declines *in vitro* or within the LCV, carbon flux from serine is redirected toward storage compound production, particularly PHB and fatty acids (James et al., 1999; Hauslein et al., 2016). To support long-term persistence in nutrient-deprived environments, *L. pneumophila* catabolizes PHB to sustain essential energy needs, contributing to long-term persistence in environmental reservoirs such as aquatic system (Gillmaier et al., 2016; James et al., 1999). This storage strategy enhances environmental resilience and may contribute to the establishment of VBNC states.

Myo-inositol represents a valuable host-derived carbon source for *L. pneumophila* because it is abundant in eukaryotic membranes, particularly within phosphoinositides such as PI, PI4P, and PI(4,5)P_2_ (Balla, 2013; Di Paolo and De Camilli, 2006). Genomic and biochemical studies have shown that *L. pneumophila* encodes a complete inositol (iol) catabolic operon enabling degradation of myo-inositol into central carbon metabolites, providing alternative carbon and energy source when amino acids become limiting (Manske et al., 2016). Disruption of the *iol* pathway significantly impairs intracellular replication in both amoebae and macrophages, demonstrating that access to this host-derived carbon pool is not merely supplemental but directly required for optimal intracellular growth (Manske et al., 2016). In parallel, nutrient liberation in the host is supported by the Type II secretion system (T2SS), which exports degradative enzymes including phosphatases, proteases, lipases, and nucleases that remodel the extracellular and vacuolar milieu (Debroy et al., 2006b; Cianciotto, 2009). Collectively, these metabolic adaptations, ranging from preferential amino acid usage, bipartite carbon metabolism, and PHB storage, to iron scavenging and host manipulation, underscore the nutritional versatility of *L. pneumophila* to adapt to the intracellular niche of protozoan and mammalian cells. This metabolic flexibility is tightly coupled to the pathogen’s biphasic life cycle and is essential to its ecological success as a facultative intracellular pathogen and resilient environmental survivor.

### 4.1 Amino acid acquisition

To support amino acid acquisition, *L. pneumophila* employs multiple strategies to increase levels of amino acids in the host. The Dot/Icm-secreted effector AnkB promotes polyubiquitination and proteasomal degradation of host proteins, thereby generating a surplus of host amino acids, which is essential for intracellular replication (Price et al., 2009; Lomma et al., 2010; Price et al., 2011). Additional effectors in the Lgt and SidE families modulate the host’s mTORC1 pathway to inhibit host protein synthesis, releasing free amino acids (De Leon et al., 2017). Transcriptomic studies during intracellular growth revealed upregulation of ABC transporters, amino acid permeases, proteases, and phospholipases that liberate and import host-derived nutrients (Bruggemann et al., 2006b). Infected human monocytic cells, Mono Mac 6 (MM6), up-regulate the neutral amino acid transporter SLC1A5, and inhibition or knockdown of SLC1A5 impairs intracellular replication of *L. pneumophila*, indicating exploitation of host amino acid import pathways (Wieland et al., 2005). In addition, *L. pneumophila* expresses its own phagosomal threonine transporter (PhtA), and deletion of *phtA* results in a threonine-auxotrophic phenotype and abrogates intracellular replication during primary murine bone marrow-derived macrophages (Sauer et al., 2005). Isotopologue profiling of bacterial and amoebal proteins confirmed that *L. pneumophila* imports and catabolizes host-derived amino acids during intracellular replication (Schunder et al., 2014; Price et al., 2011).

### 4.2 Iron acquisition

Iron acquisition is another critical metabolic adaptation to the intracellular life cycle of *L. pneumophila*, which encodes at least four distinct iron acquisition systems that allow import of ferric and ferrous iron, heme, iron-loaded peptides, and iron-loaded siderophores (Chatfield et al., 2011; Robey and Cianciotto, 2002; Viswanathan et al., 2000; O’Connell et al., 1996; Hickey and Cianciotto, 1997; O’Connor et al., 2016). It also secretes the iron-reducing pigment pyomelanin to enhance iron solubilization and uptake under limiting conditions (Chatfield and Cianciotto, 2007). Key iron acquisition genes such as lbtA (rhizoferrin biosynthesis) and feoB (ferrous iron uptake) are expressed during intracellular replication, contributing to bacterial fitness in both protozoan and mammalian hosts (Lopez et al., 2023). Although many Gram-negative bacteria energize outer-membrane uptake through the TonB-ExbB-ExbD motor, *L. pneumophila* lacks TonB homologs, the inner membrane protein that typically powers outer membrane iron transport and utilizes TonB-independent receptors such as LbtU for legiobactin uptake (Chatfield et al., 2011; Braun et al., 2023). This receptor operates with inner-membrane ABC/MFS transporters to complete iron acquisition, a configuration that represents an atypical mechanism for coupling energy to outer-membrane transport (Chatfield et al., 2011, Chatfield et al., 2012; Cianciotto, 2015). The pathogen also deploys T4SS effector MavN, a vacuolar iron transporter that localizes to the LCV and mediates iron import to sustain bacterial replication (Portier et al., 2015; Isaac et al., 2015).

## 5 Pathogenic evolution

Unicellular phagocytic amoebae are considered the evolutionary ancestors of macrophages, with highly conserved eukaryotic processes such as phagocytosis and vesicle traffic (Bozzaro and Eichinger, 2011; Cosson and Soldati, 2008). The evolutionary trajectory of *L. pneumophila* to infect macrophages has been profoundly shaped by its repeated and prolonged interactions with diverse protozoan hosts, which provide both ecological niches for replication and selective pressures that have driven the acquisition, diversification, and maintenance of an unusually large and complex arsenal of effectors (Best and Abu Kwaik, 2018; Price et al., 2024). The genome of *L. pneumophila* is highly modular and mosaic, consisting of a conserved core genome interspersed with genomic islands acquired via horizontal gene transfer (HGT) (O’Connor et al., 2011). These genomic islands often encode effector proteins, many of which contain eukaryotic-like domains, including ankyrin repeats, F-box motifs, and U-boxes, which have been acquired via interkingdom HGT from various protozoan hosts (Gomez-Valero and Buchrieser, 2019; Burstein et al., 2016; De Felipe et al., 2005; Lurie-Weinberger et al., 2010). These eukaryotic-like proteins mimic host cellular factors and are believed to enhance bacterial ability to hijack host cellular processes, such as vesicle traffic, signal transduction, and protein degradation pathways (Price et al., 2011; Weber and Faris, 2018; Hochstrasser and Hilbi, 2017). Comparative genomics of *Legionella* species further supports this model of evolution by environmental selection. Effectors that are required for replication in amoebae are often required for infection in mammalian macrophages (Park et al., 2020b). However, some amoeba-adapted effectors may have accidental effects on replication in mammalian cells, highlighting the context-specific nature of virulence and the trade-offs associated with broad host adaptability (Gomez-Valero et al., 2009; David et al., 2016; Shames et al., 2017). This evolutionary framework emphasizes that pathogenic evolution of *L. pneumophila* in humans is not the result of direct selection in human hosts, but rather a consequence of environmental adaptation to unicellular amoebae and most likely some various environmental multicellular eukaryotes. Understanding how interactions with amoebae shape pathogenic evolution to infect macrophages provides a powerful lens for dissecting pathogenic mechanisms and strategies for interrupting the transmission cycle.

## 6 Type IVB secretion system

The Dot/Icm Type IVB secretion system (T4BSS) represents one of the most extensively studied and biologically impactful secretion systems in Gram-negative intracellular pathogens. The Dot/Icm T4BSS itself likely evolved from conjugative machinery and exhibits features related to both type IVA (*Agrobacterium* VirB/D4) and type VI secretion systems (Christie and Vogel, 2000; Kubori and NAGAI, 2016). In *L. pneumophila*, the Dot/Icm T4BSS is the primary virulence determinant, governing nearly most aspects of intracellular parasitism, from host cell entry and vacuolar remodeling to immune subversion and replication within amoebae hosts and macrophages. Unlike other pathogens that encode smaller effector repertoires, *L. pneumophila* utilizes the Dot/Icm T4BSS to translocate ~350 confirmed effector proteins into host cells, which accounts for more than 11% of its genome coding capacity, making it the bacterium with the largest known effector arsenal, followed by *C. burnetiii* which harbors ~120 putative effector proteins (Crabill et al., 2018; Burstein et al., 2016; Gomez-Valero et al., 2019b; Lifshitz et al., 2013; Zhu et al., 2011). The Dot/Icm system is a biological molecular nano syringe spanning both bacterial membranes and projecting into the host cytoplasm via contact with the LCV membrane (Ghosal et al., 2017; Durie et al., 2020; Bock et al., 2021).

The Dot/Icm apparatus comprises ~27 essential components encoded by *dot* (defective in organelle trafficking) and *icm* (intracellular multiplication) genes, and the apparatus is organized into functional subcomplexes that coordinate substrate recognition, energy generation, and effector translocation (Prashar and Terebiznik, 2015; Bock et al., 2021; Andrews et al., 1998; Berger and Isberg, 1993; Berger et al., 1994; Brand et al., 1994; Purcell and Shuman, 1998; Segal and Shuman, 1997; Vogel et al., 1998). The core transmembrane complex includes DotC, DotD, DotF (IcmG), DotG (IcmE), and DotH (IcmK), which form the secretion channel through both the inner membrane and outer membrane (Gomez-Valero et al., 2019a; Vincent et al., 2006; Buscher et al., 2005; Gomis-Ruth et al., 2002; Sutherland et al., 2012). DotC amd DotD are crucial for localization of DotH to the OM, while DotF is responsible for energy transduction through DotG (Sutherland et al., 2012; Gomis-Ruth et al., 2002; Vincent et al., 2006; Buscher et al., 2005). These components assemble to form a stable channel structurally characterized by a central secretion chamber with 13-fold symmetry and a funnel-like architecture (Vincent et al., 2006; Ghosal et al., 2019a; Chetrit et al., 2018; Park et al., 2020a). A second cytoplasmic subcomplex comprises DotL (IcmO), DotM (IcmP), DotN (IcmJ), and the chaperones IcmS, IcmW, and LvgA (Gomez-Valero et al., 2019a; Vincent et al., 2006; Buscher et al., 2005; Gomis-Ruth et al., 2002; Sutherland et al., 2012). These proteins constitute the coupling complex responsible for substrate recognition and delivery to the translocation channel (Meir et al., 2018; Nagai and Kubori, 2011; Ninio et al., 2005; Vincent et al., 2012; Cambronne and Roy, 2007). DotL is a Type IV coupling protein (T4CP) with nucleotide-binding and α-helical domains, providing ATPase activity essential for energizing substrate export (Sutherland et al., 2012; Vincent et al., 2012; Buscher et al., 2005; Gomis-Ruth et al., 2002). Additional subunits, including DotU, IcmF, DotK, IcmR, and IcmQ, contribute to polar localization, assembly fidelity, and structural stabilization of the apparatus (Bock et al., 2021; Gomez-Valero et al., 2019a; Vincent et al., 2006; Gomis-Ruth et al., 2002; Buscher et al., 2005). The apparatus is anchored at one pole of the bacterium, and its proper localization is necessary for LCV formation and host cell modulation (Jeong et al., 2017; Berger and Isberg, 1993; Purcell and Shuman, 1998).

Protein substrates are generally targeted to the secretion system via C-terminal translocation signals that are located at the last ~20–35 amino acids, which often contain glutamic acid-rich E-block motifs or other signals that bind the chaperone IcmSW (Lifshitz et al., 2013; Nagai et al., 2005; Kubori et al., 2008; Burstein et al., 2009; Huang et al., 2011; Cambronne and Roy, 2007). Effector delivery is highly dynamic and temporally regulated through cyclic-di-GMP signaling, with Lpl0780/Lpp0809 and diguanylate cyclase enzymes modulating effector release kinetics (Allombert et al., 2021). Some effectors are injected upon bacterial contact with the host cell plasma membrane, while others are injected by intra-vacuolar bacteria (Dumenil et al., 2004; Conover et al., 2003; Charpentier et al., 2009; Liu et al., 2008). Translocation of AnkB by attached extracellular bacteria upon contact with human macrophage and *A. polyphaga* serves as an example of translocation upon bacterial contact to the host plasma membrane (Price et al., 2009). Some substrates such as SidM/DrrA and the SidE family are translocated early in the LCV and disappear as infection progresses, while others like LepB remain associated with the LCV throughout (Ingmundson et al., 2007; Neunuebel et al., 2011; Bardill et al., 2005). This temporal hierarchy aligns with the temporal functional requirements during the infection cycle, including Rab1 activation and deactivation via SidM, SidD, and LepB at various stages of the infection (Neunuebel et al., 2011).

### 6.1 Biogenesis of the LCV in amoebae hosts and macrophages

*L. pneumophila* has evolved mechanisms to evade amoebae predation and persist intracellularly, evolving from a prey into a predator, becoming a facultative intracellular bacterium. Following uptake by amoebae, the Dot/Icm Type IV Secretion System of *L. pneumophila* functions as a biological nano-syringe to inject ~350 different effector proteins into the host cell (Hubber and Roy, 2010; Prashar and Terebiznik, 2015; Nagai and Kubori, 2011). *L. pneumophila* utilizes these effector proteins to intercept ER secretory vesicles to remodel its phagosome into an ER-derived vacuole that evades the endosomal-lysosomal degradation pathway, which is designated as the LCV (Price et al., 2024; Kawabata et al., 2021; Horwitz, 1983a; Isberg et al., 2009; Horwitz, 1983b; Fields et al., 2002; Swanson and Hammer, 2000; Swanson and Sturgill-Koszycki, 2000; Oliva et al., 2018) (**Figure 1**). Following ~4-hour lag phase within the LCV, bacteria replicate every ≈60 minutes until the host cell is either lysed or bacteria are exocytosed by the amoeba into the environment (Gao and Abu Kwaik, 1999a, GaoAbu Kwaik, 1999b; Hägele et al., 1998). A full intracellular cycle of LCV remodeling, intracellular replication, and release following phagocytosis is highly orchestrated event that occurs within ≈18 hours under controlled laboratory condition (Hägele et al., 1998; Amer and Swanson, 2002; Molmeret et al., 2004; Molofsky and Swanson, 2004). The length of the life cycle in various environments is likely to be different from laboratory conditions.

Once inhaled, *L. pneumophila* is taken up by alveolar macrophages (Oliva et al., 2018; Fields et al., 2002; Price et al., 2024; Bozue and Johnson, 1996; Horwitz, 1984). Similar to the protozoan hosts, *L. pneumophila* coordinates delivery of the ~350 effectors to establish the vacuole which subverts lysosomal fusion and intercepts ER-to-Golgi vesicle traffic, to become an ER-derived LCV (Swanson and Sturgill-Koszycki, 2000; Ku et al., 2012; Fields et al., 2002; Swanson and Isberg, 1995; Horwitz, 1983b; Wilson et al., 1994; Tisdale et al., 1992) (**Figure 1**). Among the earliest events of LCV biogenesis is recruitment of secretory vesicles derived from the ER and ER-Golgi intermediate compartment (ERGIC), a process initiated in part by the guanine-exchange factor RalF, which activates host Arf1 to promote docking and fusion of ER-derived vesicles to the LCV (Nagai et al., 2002). A central regulatory node in LCV biogenesis is Rab1, a master GTPase governing ER-Golgi anterograde trafficking (Ingmundson et al., 2007). Rab1 is targeted on the LCV membrane through a temporally controlled effector cascade. The SidM (DrrA) effector on the LCV membrane recruits and activates Rab1 while locking it in an active state through non-canonical AMPylation (Muller et al., 2010; Kagan and Roy, 2002). The GTP-bound Rab1 is stabilized by LidA by preventing its extraction from membranes, amplifying vesicle tethering and ER factor recruitment (Neunuebel et al., 2012). Inactivation of Rab1 signaling is equally critical for vacuole maturation and is mediated by SidD, a specific de-AMPylase that restores Rab1 to conventional regulatory cycling, followed by LepB, a Rab1 GAP that drives GTP hydrolysis to complete Rab1 inactivation and progression of the trafficking program (Neunuebel et al., 2012; Tan and Luo, 2011; Mishra et al., 2013).

Parallel to diverting early secretory traffic, *L. pneumophila* actively blocks endosomal maturation and retrograde fusion events that would otherwise lead to vacuole acidification and lysosomal delivery. The VipD effector, a unique patatin-like phospholipase, selectively hydrolyzes phosphatidylinositol lipids on endosomal membranes and functionally antagonizes Rab5 and Rab22, two GTPases required for early endosome motility and fusion, thereby preventing LCV interception by the canonical endocytic pathway (Ku et al., 2012; Gaspar and Machner, 2014). In addition, RidL directly binds vacuolar protein sorting protein (VPS29), a core retromer subunit essential for endosome-trans-Golgi retrieval, disabling retromer assembly and hindering retrograde vesicle exchange with lysosomal or Golgi-connected compartments that could compromise vacuolar integrity (Finsel et al., 2013; Romano-Moreno et al., 2017). Collectively, these effectors create a LCV that simultaneously mimics an ER-secretory vesicle/organelle while being insulated from degradative trafficking.

The ability of *L. pneumophila* to replicate within a broad range of hosts, from freshwater amoebae to human macrophages, is further supported by its capacity to modulate host cell responses beyond phagosome remodeling, such as manipulation of autophagy, host sphingolipid metabolism, and NF-κB and various innate immune responses (Choy et al., 2012; Rolando et al., 2016). Thus, the intracellular life cycle of *L. pneumophila* in macrophages reflects a successful evolutionary repurposing of mechanisms initially developed to colonize protozoa, representing a striking case of environmental selection pressures shaping pathogenesis in an accidental human host. However, modulation of mammalian-specific processes that are absent in unicellular amoebae indicate adaptation of *L. pneumophila* to unknown multicellular eukaryotes prior to human infections.

### 6.2 The perplexing dispensability of most effectors for intracellular replication

Genetic screens and bioinformatics have revealed that many Dot/Icm substrates contain eukaryotic-like domains, suggesting their acquisition via interkingdom HGT from amoebal hosts or their endosymbionts (Gomez-Valero et al., 2019b; Cazalet et al., 2004; Franco et al., 2009; Lurie-Weinberger et al., 2010). Phylogenetic analyses date the last common ancestor of *Legionella* (LLCA) to ~1.89 billion years ago, indicating early co-evolution with protozoan hosts upon the emergence of eukaryotes (Hugoson et al., 2022). The combination of intra- and inter-kingdom HGT, competence of natural DNA transformation by *L. pneumophila*, and adaptation to various environmental host species has resulted in acquisition of remarkable genome plasticity, including more than 18,000 putative effector genes across the *Legionella* genus (Gomez-Valero et al., 2019b). These effectors are essentially a toolbox for *L. pneumophila* where certain effectors are needed in a certain host but not others (Best and Abu Kwaik, 2018; Price et al., 2024).

Despite the vast effector repertoire, deletion of most individual effectors often fails to result in a detectable defective phenotype in intracellular replication. Even deletion of 31% of known effectors results in only modest replication defects (O’Connor et al., 2011). A possible reason for this is redundancy, which can be explained by overlaps in pathways, targets, cellular functions, and even molecular mechanisms (Ghosh and O’Connor, 2017). Effector redundancy in *L. pneumophila* most likely reflects the adaptation to diverse protozoan hosts (Price et al., 2024; Best and Abu Kwaik, 2018). The SidE family (SidE, SdeA, SdeB, SdeC) exemplifies this redundancy. These effectors are dispensable when deleted individually, but collectively they are essential for robust intracellular replication due to their role in ubiquitinating host proteins like Reticulon 4 and Rab33b (Luo and Isberg, 2004; Kotewicz et al., 2017; Qiu et al., 2016; Vanrheenen et al., 2006). Functions of *L. pneumophila* effectors have been discussed in recent reviews (Graham et al., 2024; Romanov and O’connor, 2024).

### 6.3 Core effectors of *L. pneumophila*

Comparative genomics analyses indicate that among the ≈18,000 effectors in *Legionella* species, only 9 core effectors are conserved across all 59 sequenced *Legionella* species, and these are MavN, VipF, RavC, CetLp1, Lpg2832, Lpg3000, AnkH/LegA3, LceA, and LceB (Wexler et al., 2022; Burstein et al., 2016; Gomez-Valero et al., 2019b). This underscores the existence of a small subset of effectors that likely perform non-redundant, indispensable functions across diverse ecological niche in all *Legionella* species (Best and Abu Kwaik, 2018). Strikingly, several of these core effectors are also conserved in *Coxiella* and *Rickettsiella*, which are phylogenetically related to *Legionella* and encode a functional Dot/Icm system, despite having diverged from *Legionella* ≈1.89 billion years ago (Hugoson et al., 2022). This evolutionary conservation indicates that the Dot/Icm system and a minimal number of core effector toolkit were already established prior to the radiation of modern *Legionella* species ≈1.89 billion years ago and were retained in *Coxiella* and *Rickettsiella* that adapted to distinct intracellular lifestyles. Among these conserved effectors, MavN mediates iron acquisition by the LCV, and its homologs are also present in *C. burnetii* and *R. grylli* (Isaac et al., 2015; Christenson et al., 2019). Similarly, VipF, a tandem GNAT-family acetyltransferase with two catalytic GNAT domains, targets the host translation factor eIF3K, modulating translation inhibition (Syriste et al., 2024). The AnkH core effector is also conserved in *C. burnetii* and *R. grylli* and interacts with the highly conserved host LARP7 components of the host 7SK snRNP complex, thereby interfering with transcription elongation (Burstein et al., 2016; Gomez-Valero et al., 2019b). Together, these findings indicate that core effectors target highly conserved eukaryotic processes, such as nutrient acquisition, transcriptional regulation, and protein synthesis. The host target and biological function of other core effectors are yet to be discovered.

### 6.4 Metaeffectors of *L. pneumophila*

*L. pneumophila* encodes a specialized regulatory layer of “metaeffectors”, which are bacterial effectors that post-translationally control the activity, localization, or stability of other Dot/Icm effectors, thereby preserving temporal precision and functional inactivation of effector activity. A hallmark example is SidJ, a calmodulin-dependent polyglutamylase that modifies the catalytic glutamate residue of the SidE family, inhibiting NAD-dependent ubiquitin ligase activity once Rab1 recruitment and ER remodeling are established on the LCV (Black et al., 2019; Gan et al., 2019b). This delayed translocation of the SidJ metaeffector ensures that SidE effectors function temporarily and transiently during early vacuole biogenesis but are subsequently inactivated to prevent excessive ubiquitin signaling (Black et al., 2019; Gan et al., 2019b). Similarly, the E3 ubiquitin ligase LubX metaeffector targets the effector SidH for K48-linked ubiquitination and subsequent proteasomal degradation, directly controlling effector abundance and mitigating SidH-associated toxicity during later stages of infection (Kubori et al., 2010). Another regulatory axis involves MesI, a metaeffector that binds to the glycosyl hydrolase SidI, suppressing its protein synthesis inhibitory activity to prevent premature translational arrest and host cell death (Joseph et al., 2020).

Additional metaeffector-effector pairs further illustrate the diversity of regulatory strategies employed by *L. pneumophila.* The de-ubiquitinase LupA metaeffector counteracts ubiquitin-dependent toxicity of the SNARE-mimic effector LegC3, while the ankyrin-repeat protein AnkJ metaeffector suppresses the translation- and actin-inhibitory effector SidL through direct binding (Urbanus et al., 2016; Machtens et al., 2025; Joseph and Shames, 2021). Likewise, the SidP metaeffector binds and suppresses the phosphoinositide kinase MavQ independently of its own PI3P phosphatase activity, highlighting that metaeffectors may regulate effectors through non-enzymatic mechanisms (Urbanus et al., 2016; Joseph and Shames, 2021). Collectively, these regulatory circuits ensure that effector functions are restricted to precise temporal windows during infection, avoiding fatal damage to the replication niche. Indeed, temporal translocation profiling demonstrates that early effector delivery is dominated by host permissiveness, while late-stage effectors and metaeffectors dampen earlier virulence programs to restore host homeostasis and permit bacterial egress (Kubori et al., 2010; Jeong et al., 2015; Qiu and Luo, 2017a).

### 6.5 Para-effectors of *L. pneumophila*

Beyond classical effector redundancy and the hierarchical regulation imposed by effector metaeffectors pairs, *L. pneumophila* deploys sets of para-effectors, which are independently translocated effectors that target the same host protein target of the bacterial effector through distinct biochemical mechanisms. This is an additional layer for robust control of key host processes to remodel diverse protozoan hosts into proliferative niches. A well-characterized example of host target modulated by para-effectors is the manipulation of the Rab1 GTPase cycle by para-effectors (SidM, SidD, LepB, AnkX and Lem3). The Rab1-directed para-effector module consists of multiple translocated effectors that modify Rab1 through distinct biochemical mechanisms. The para-effector SidM activates and AMPylates Rab1 to promote ER-derived vesicle recruitment, whereas the de-AMPylase para-effector SidD removes this modification to terminate Rab1 signaling (Ingmundson et al., 2007; Muller et al., 2010; Neunuebel et al., 2011). In parallel, para-effector LepB functions as a Rab1 GAP to accelerate GTP hydrolysis, while para-effector AnkX phosphocholinates Rab1, and the counteracting Lem3 subsequently removes this modification (Ingmundson et al., 2007; Mukherjee et al., 2011; Tan et al., 2011). Although these Rab1-targeting effectors do not directly regulate one another, unlike metaeffectors, SidM, SidD, LepB, AnkX, and Lem3 collectively function as para-effectors that modify host targets, ensuring that Rab1 cycling is tightly controlled even under heterogeneous host conditions.

Similar para-effector networks shape phosphoinositide homeostasis on the LCV. The PI(4)P-binding ubiquitin ligases SidC/SdcA, the PI(3)P-binding protein LpnE, the dual PI phospholipase VipD, and the phosphoinositide phosphatase SidF all are para-effectors that act independently yet converge on stabilizing ER-like membrane identity and evading endosomal-lysosomal fusion of the LCV (Hsu et al., 2014; Weber et al., 2006; Gaspar and Machner, 2014). In addition, parallelism also exists in ubiquitin signaling. The ubiquitin-modulating six para-effectors LegU1, AnkB, MavC, MvcA, LegK1, and RavZ each reshape ubiquitin-mediated regulation through biochemically distinct modification of host targets (Kubori et al., 2008; Price et al., 2009; Gan et al., 2019a, Gan et al., 2020; Ge et al., 2009; Choy et al., 2012).

A distinct form of para-effector pair is shown by the chromatin-modifying effectors RomA and LphD (Schator et al., 2023). RomA, a SET-domain methyltransferase, tri-methylates histone H3 at lysine 14 (H3K14), while LphD is a eukaryotic-like histone deacetylase that specifically removes H3K14 acetylation, thereby enabling RomA-mediated methylation (Schator et al., 2023). Unlike other para-effector networks, RomA and LphD display strong functional interdependence that arises from sequential modification of the same host residue rather than direct effector-effector regulation. Loss of either effector impairs intracellular replication of *L. pneumophila* during infection of THP-1 macrophages and *A. castellanii*, whereas a double knockout strain partially restores bacterial growth defect, defining a unique para-effector relationship based on cooperative chromatin remodeling (Schator et al., 2023). Together, these para-effector systems enable *L. pneumophila* to stabilize key host trafficking, membrane identity, ubiquitin signaling, and chromatin states that are required for LCV maturation and sustained intracellular replication to adapt to diverse protozoan reservoirs and mammalian macrophage hosts.

## 7 Type II secretion system

Despite the conservation of T2SS architecture among Proteobacteria, the output and substrate repertoire of the system vary markedly between species. Some substrates appear to be conserved across the genus and are thought to have arisen in a common ancestor of *Legionella* and its closest relative *Aquicella* (White and Cianciotto, 2019). This mosaic evolutionary origins likely reflects the selective pressures exerted by the intracellular environment and host-pathogen interactions over a long evolutionary time. Although T2SSs are not universal among Gram-negative bacteria, they are widely distributed among the γ-Proteobacteria and found in both animal and plant pathogens, including *Pseudomonas aeruginosa*, *Klebsiella pneumoniae*, *Yersinia enterocolitica*, *Vibrio cholerae*, and *Erwinia amylovora* (Cianciotto and White, 2017; White and Cianciotto, 2019; Jyot et al., 2011; Yang et al., 2023; Von Tils et al., 2012; Reichow et al., 2010; Zhao et al., 2005). In these organisms, T2SSs facilitate a broad array of virulence-related functions, such as tissue degradation, biofilm formation, evasion of host defenses, and host colonization. Interestingly, despite the vast functional repertoire of T2SS, they have not been implicated in interbacterial competition. Unlike the Type VI Secretion System (T6SS), which is used for contact-dependent killing of competing bacteria, the T2SS appears specialized for secretion into the extracellular milieu or host interface, emphasizing its roles in environmental adaptation and host manipulation rather than bacterial antagonism (White and Cianciotto, 2019; Shaliutina-Loginova et al., 2023; Naskar et al., 2021; Cianciotto and White, 2017).

The T2SS is a multi-protein complex and plays a pivotal role in environmental persistence, adaptation to host cells, and pathogenesis of *L. pneumophila* (Korotkov et al., 2012; Cianciotto, 2009). Evolutionarily related to the type IV pilus machinery, the T2SS operates as a sophisticated two-step secretion system that translocates folded proteins across the cytoplasmic and outer membranes into the extracellular space (Korotkov et al., 2012; Korotkov and Sandkvist, 2019; Ghosal et al., 2019b; White et al., 2019; White and Cianciotto, 2019; Rossier et al., 2008, Rossier et al., 2004; Soderberg et al., 2008; White and Cianciotto, 2016; Cianciotto and White, 2017; Debroy et al., 2006b; Galka et al., 2008; Truchan et al., 2017; Cianciotto, 2009). The T2SS apparatus in *L. pneumophila* consists of 12 core proteins (T2S C, D, E, F, G, H, I, J, K, L, M, and O), which assemble into four functional subcomplexes (White and Cianciotto, 2019; Ghosal et al., 2019b; Cianciotto and White, 2017; Peabody et al., 2003). The first subcomplex is an outer membrane secretin, which is a multimeric ring of D proteins that forms the final secretion pore in the outer membrane. This channel serves as the exit site for mature substrates destined for the extracellular environment or bacterial surface (Ghosal et al., 2019b). The second is an inner membrane platform, composed of F, L, and M proteins that are anchored in the inner membrane, spanning the periplasmic space, and acts as a scaffold for the entire secretion system. This platform interfaces directly with the secretin and provides docking sites for other subunits, including the ATPase motor (Ghosal et al., 2019b). The third is a periplasmic pseudopilus that functions analogously to a piston or Archimedean screw. This pilus-like structure is composed of a major pseudopilin (G) and minor pseudopilins (H, I, J, and K) (Cianciotto, 2005; Ghosal et al., 2019b; Cianciotto and White, 2017). It undergoes polymerization-driven extension to push substrates through the secretin pores. The O protein cleaves and methylates pseudopilin precursors prior to their incorporation into the pseudopilus, facilitating precise assembly and function (Cianciotto, 2005; Ghosal et al., 2019b; Cianciotto and White, 2017; Peabody et al., 2003). The fourth is a cytoplasmic ATPase, which is a hexamer of E component recruited to the inner membrane platform. It hydrolyzes ATP to provide energy necessary for pseudopilus extension and substrate translocation. The activity of this ATPase is essential for the dynamic motion of the piston mechanism (Ghosal et al., 2019b; White and Cianciotto, 2019). Coupling of the outer membrane and inner membrane complexes is mediated by the clamp C protein component, which also contributes to substrate recognition by coordinating substrate handoff from the periplasm to the pseudopilus machinery (Ghosal et al., 2019b; White and Cianciotto, 2019). The secretion process begins with substrate recognition via the Sec or Tat translocon pathways, which transport unfolded proteins bearing N-terminal signal peptides across the inner membrane into the periplasm (Tsirigotaki et al., 2017; Berks, 2015). There, the signal peptides are cleaved, allowing the substrate proteins to fold into their active tertiary conformation. These folded proteins are then recognized by the T2SS apparatus (Korotkov and Sandkvist, 2019; Pineau et al., 2021).

There are approximately 120 proteins that are predicted to be secreted via the T2SS in *L. pneumophila*, with at least 27 confirmed substrates displaying over 20 distinct enzymatic activities, including lipases, proteases, glycosidases, nucleases, and phosphatases (Adams et al., 2025; Rossier et al., 2004; Cianciotto and White, 2017; White et al., 2018; Cianciotto, 2009; Debroy et al., 2006b; Debroy et al., 2006a). These enzymes contribute to the degradation of host macromolecules, nutrient acquisition, and remodeling of the vacuolar niche. Several substrates also exhibit structural and functional mimicry of eukaryotic proteins, suggesting evolutionary adaptation to intracellular life. For example, ProA is a zinc metalloprotease that degrades complement proteins and extracellular matrix components, promoting tissue invasion and immune evasion (Scheithauer et al., 2021, Scheithauer et al., 2022, Scheithauer et al., 2025). ChiA is a chitinase that enhances lung persistence, likely by degrading mucins or host glycoproteins (Rehman et al., 2020). PlaC is an acyltransferase involved in phospholipid modification (White et al., 2018). SrnA is a ribonuclease with potential roles in modulating host RNA stability (Rossier et al., 2009). NttA and NttC are novel T2SS-secreted proteins required for optimal infection of certain amoebae species (Tyson et al., 2014). Many of these substrates, including ProA and ChiA, localize outside the LCV and form distinctive ring-like patterns surrounding the vacuole membrane in host cells, implying they function in modifying the host cytosolic environment or vacuolar interface (Truchan et al., 2017; White and Cianciotto, 2016).

The T2SS is crucial for ability of *L. pneumophila* to infect both amoebae hosts and mammalian cells, form biofilms, modulate immune responses, and survive in aquatic environments (Rossier et al., 2008, Rossier et al., 2004; Soderberg et al., 2008; White and Cianciotto, 2016; Cianciotto and White, 2017; Debroy et al., 2006b; Mccoy-Simandle et al., 2011; Mallama et al., 2017). Studies have shown that mutants deficient in T2SS components are severely impaired in their ability to replicate within amoebae such as *A. castellanii* and *V. vermiformis* (Tyson et al., 2013). Other studies have also shown that T2SS is essential in intracellular replication of macrophages (White and Cianciotto, 2016). These impairments underscore the central role of the T2SS in facilitating intracellular survival and replication. Beyond intracellular infection, the T2SS contributes significantly to *L. pneumophila*’s environmental resilience, as it plays roles in sliding motility, biofilm formation, and survival in water at low temperatures (4-25 °C) (Soderberg et al., 2008; Soderberg and Cianciotto, 2008; Duncan et al., 2011; Stewart et al., 2009).

## 8 The innate immune response to *L. pneumophila*

The innate immune system is the first line of defense against *L. pneumophila*, and this response is multifaceted, involving several pattern recognition receptors (PRRs) (Schuelein et al., 2011; Massis and Zamboni, 2011). Although *L. pneumophila* has evolved an extensive effector repertoire while adapting to protozoan hosts, these adaptations were not shaped by sustained selection to evade vertebrate PRR, and as a result the bacterium remains susceptible to PRR-mediated restriction in mammalian macrophages (Brown et al., 2017; Grigoryeva and Cianciotto, 2021). This is particularly evident in murine models, which does not occur in humans. For instance, while A/J mice is the only inbred strain permissive to *L. pneumophila*, all other inbred strains such as C57BL/6 and BALB/c are non-permissive to infection due to rapid pyroptotic death of macrophages (Susa et al., 1998; Akamine et al., 2007; Coers et al., 2007; Deng et al., 2001; Tateda et al., 1998; Fernandez-Serrano et al., 2003; Dietrich et al., 1995; Diez et al., 2003). Flagellin of *L. pneumophila* is sensed by the cytosolic neuronal apoptosis inhibitory protein 5 (Naip5), which forms a complex with NLRC4 to activate the inflammasome (Dietrich et al., 1995; Diez et al., 2003; Wright et al., 2003; Fortier et al., 2007). This Dot/Icm-dependent sensing mechanism results in caspase-1 activation, pyroptotic cell death, and the release of proinflammatory cytokines such as IL-1β and IL-18 (Mascarenhas et al., 2017; Case and Roy, 2011).

Caspase-7 is activated downstream of canonical inflammasome signaling during *L. pneumophila* infection and functions as a downstream executioner that reinforces macrophage death and bacterial restriction. NLRC4-dependent caspase-1 activity leads to caspase-7 activation during *L. pneumophila* infection, and loss of caspase-7 increases mice permissiveness to bacterial replication in mouse macrophages (Akhter et al., 2009). Combined loss of gasdermin-D (GSDMD) and caspase-7 in mice phenocopies NLRC4 deficiency in permitting replication, indicating that caspase-7 acts in parallel with gasdermin-mediated pyroptosis to enforce robust restriction (Goncalves et al., 2019). Thus, caspase-7 should be viewed as a downstream amplifier of inflammasome-driven cell death and inflammation that collaborates with, but is mechanistically distinct from, caspase-1 and caspase-11 pathways in shaping the macrophage response to *L. pneumophila* in mice.

In mammalian macrophages, *L. pneumophila* activates the non-canonical inflammasome through an interferon-licensed pathway in which the guanylate-binding proteins (GBPs) destabilize the LCV, allowing cytosolic exposure of bacterial LPS and subsequent activation of caspase-4/5 in humans/caspase-11 in mice (Aachoui et al., 2015; Bass et al., 2023). Signaling through TRIF and IFN-dependent pathways induces transcriptional upregulation of pro-caspase-11, licensing cells for non-canonical inflammasome activation in mice (Rathinam et al., 2012; Aachoui et al., 2015). IFN-inducible guanylate-binding proteins (GBPs) are rapidly mobilized to pathogen-containing vacuoles to destabilize vacuolar membranes, which are essential for cytosolic LPS surveillance in infected cells that lead to a second pathway of pyroptosis (Aachoui et al., 2013; Case et al., 2013; Pilla et al., 2014; Price et al., 2019; Aachoui et al., 2015)-. Upon infection, IFN-induced GBPs are recruited to intracellular pathogen vacuoles, including the LCV, where they promote vacuolar membrane disruption and facilitate inflammasome access to bacterial products (Bass et al., 2023; Feeley et al., 2017). Disruption of LCV integrity enables bacterial LPS to enter the host cytosol, where it directly binds and activates caspase-11, independently of TLR4 signaling (Hagar et al., 2013; Kayagaki et al., 2011). Activated caspase-11 cleaves GSDMD, triggering pore formation in the host membrane and pyroptotic cell death, which can synergize with secondary NLRP3–caspase-1 activation to amplify IL-1β release (Shi et al., 2015; Kayagaki et al., 2015). Thus, in IFN-primed macrophages in both mice and humans, caspase-11 and caspase-4 activation during *Legionella* infection is driven not by a dedicated effector-triggered mechanism, but by GBP-dependent destabilization of the LCV that permits cytosolic exposure of LPS. In addition to mice caspase-11- and human caspse-4-driven non-canonical inflammasome activation, downstream executioner caspases further enforce macrophage cell death during infection.

### 8.1 Opposing effects of *Legionella* effectors on host translation

Despite the reported global reprogramming of the host transcriptome, at least in part by AnkH and RomA nucleomodulins of *L. pneumophila*, multiple effectors, including RavX, SidI, SidL, LegK4, and the Lgt family (Lgt1, Lgt2, and Lgt3), inhibit host protein translation by targeting elongation factors and ribosomes (Von Dwingelo et al., 2019; Rolando et al., 2013, Rolando et al., 2023; Lockwood et al., 2022; Copenhaver et al., 2014, Copenhaver et al., 2015). However, several lines of evidence from various research groups demonstrate that this translational blockade is not global (Lockwood et al., 2022; Price et al., 2020; Shames et al., 2017; Khweek et al., 2013; Fontana et al., 2011; Barry et al., 2017; Fontana et al., 2012; Shin et al., 2008; Abu-Zant et al., 2007; Losick et al., 2010; Asrat et al., 2014; Losick and Isberg, 2006; Bartfeld et al., 2009; Liu et al., 2020; Copenhaver et al., 2015; Casson et al., 2017; Mascarenhas et al., 2015; Barry et al., 2013). Unlike a universal translational arrest, selective host proteins continue to be translated and upregulated.

Strikingly, several *L. pneumophila* effectors themselves counteract the translational blockade to actively promote production of host immune proteins. The LegA9 and LegC4 effectors augment translation of pro-inflammatory cytokines, while the LamA effector drives robust M1 pro-inflammatory polarization through up-regulation of host glycolysis and induction of inflammatory mediators (Lockwood et al., 2022; Price et al., 2020; Shames et al., 2017; Khweek et al., 2013). In addition, macrophages infected with *L. pneumophila* also undergo enhanced translation of IL-1α and IL-23α, along with sustained activation of MAPK signaling and both canonical and non-canonical NF-κB pathways (Lockwood et al., 2022; Fontana et al., 2011; Barry et al., 2017; Fontana et al., 2012; Shin et al., 2008; Abu-Zant et al., 2007; Losick et al., 2010; Asrat et al., 2014; Losick and Isberg, 2006; Bartfeld et al., 2009). Consistent with this, IL-1α is selectively upregulated, which triggers the cells, leading to activation of bystander myeloid cells (Asrat et al., 2014; Liu et al., 2020; Copenhaver et al., 2015; Casson et al., 2017; Barry et al., 2017; Mascarenhas et al., 2015; Barry et al., 2013).

In addition, ribosomal perturbation caused by translational inhibition activates stress kinases and ribotoxic stress responses that function as danger signals to amplify immunity (Fontana et al., 2011). This phenomenon, whereby pathogen-encoded effectors stimulate host defense rather than suppress it, aligns with the emerging paradigm of Effector-Triggered Immunity (ETI) (Fontana et al., 2011; Subramanian et al., 2023). Deletion of ETI-inducing effectors such as LegA9, LegC4, LamA results in increased bacterial replication in macrophages and murine infection models, demonstrating that these “virulence” factors paradoxically serve host-protective roles during infection (Shames et al., 2017; Park et al., 2020b). It is possible that these effectors are amoebae-adapted effectors with accidental effects on human macrophages.

Although complete translational shutdown in eukaryotic cells typically induces autophagy, cellular dysfunction, and cell death within hours, *L. pneumophila*-infected macrophages remain viable for 24–72 hours, indicating that a substantial subset of host proteins continues to be translated to maintain cellular integrity (Acevo-Rodriguez et al., 2020; Dang and Back, 2021). This supports a model in which translational regulation during infection is highly and selectively modulated, rather than globally repressed. The precise temporal and spatial control of this antagonistic effector network remains unresolved, but it is clear that host translation is both selectively suppressed and selectively activated (Lockwood et al., 2022; Asrat et al., 2014; Belyi et al., 2008). Collectively, the data indicate that *L. pneumophila* does not trigger global translational arrest, but instead rewires host translation through competing effector functions, some enforcing inhibition, others restoring or enhancing translation of certain proteins. This places *L. pneumophila* alongside *Pseudomonas syringae* as a key model for studying ETI, where bacterial effectors operate simultaneously as virulence factors and innate immune agonists, shaping the outcome of infection through a dynamic, antagonistic regulatory network (Block and Alfano, 2011).

### 8.2 Authophagy and mitochondrial immune checkpoint interference

Autophagy is another arm of the host defense system that *L. pneumophila* must circumvent, and mitochondrial physiology is critical upstream regulator of this pathway (Amer and Swanson, 2005; Choy et al., 2012; Horenkamp et al., 2015; Thomas et al., 2020). While some effectors trigger autophagic recognition, like LegA9, others actively inhibit the process (Khweek et al., 2013). The RavZ effector cleaves lipidated Atg8/LC3, thereby irreversibly blocking autophagosome maturation and fusion with lysosomes (Choy et al., 2012). This not only prevents bacterial degradation but may also limit the presentation of bacterial antigens to the adaptive immune system.

In addition, the pathogen manipulates mitochondrial function through effectors that either modulate mitochondrial dynamics or directly alter respiration to suppress autophagy. Mitochondrial stress signals, such as loss of membrane potential, reactive oxygen species (ROS), and energy imbalance, are major triggers for AMPK-ULK1 autophagy induction and PINK1-Parkin mitophagy (Kim et al., 2011; Jin and Youle, 2012; Zhou et al., 2022). Mitochondrial function is regulated by ADP-ribosylation via the Ceg3 and Lpg0081 effectors, which modulate the host ADP/ATP translocase (Fu et al., 2022). The actin cytoskeleton is manipulated via proteins that target Arp2/3 and microtubules, further enhancing pathogen survival and replication (Michard et al., 2015). The Lpg0080 and Lpg0081 effectors function as ADP-ribosyl transferases and hydrolases targeting the mitochondrial ADP/ATP translocase, modulating energy availability and suppressing mitochondrial immunity (Kubori et al., 2022; Fu et al., 2022). By dampening mitochondrial-driven autophagy signals and simultaneously blocking LC3-mediated autophagosome maturation, *L. pneumophila* effectively cripples both autophagy initiation and execution, reinforcing intracellular survival while minimizing mitochondrial-encoded danger signals that could amplify inflammation.

### 8.3 Role of neutrophil in early host defense

While *L. pneumophila* preferentially infects macrophages, neutrophils are essential to the early innate immune response. Neutrophils are heavily recruited to the lungs in experimental models in guinea pigs and A/J mice (Davis et al., 1983; Susa et al., 1998; Akamine et al., 2007; Coers et al., 2007; Deng et al., 2001). Histologically, this is reflected by intense intra-alveolar inflammation, with polymorphonuclear leukocytes, macrophages, and necrotic debris filing the alveolar spaces (Lattimer et al., 1979; Tateda et al., 2001). Mechanistic studies in mouse models have shown that IL-1 receptor-MyD88 signaling and NOD1/2 pathways drive chemokine production and neutrophil recruitment, and that this recruitment is required for efficient pulmonary clearance of *L. pneumophila* (Frutuoso et al., 2010; Mascarenhas et al., 2015). Neutrophils, along with alveolar macrophages, are among the first cells to receive Dot/Icm-translocated effectors *in vivo* and constitute major early sources of TNF and IL-1α, positioning them as both an intracellular niche and a key effector population during acute infection (Copenhaver et al., 2014). In A/J mice, neutrophils cooperate with monocytes and innate lymphocytes to produce IL-12 and IFN-γ, shaping a protective cytokine milieu that restricts bacterial replication (Casson et al., 2017). Consistent with this, neutrophil-derived ROS and TNF are essential for early control of lung infection in resistant mouse strains (Ziltener et al., 2016). Whether these events occur in human neutrophils remains unknown.

More recent work has clarified that neutrophils rapidly restrict *L. pneumophila* in a Dot/Icm-dependent manner (Price et al., 2021). Human neutrophils respond to the T4SS-injected LamA effector with upregulated glycolysis, granule fusion to the *Legionella*-containing phagosome (LCP), and ROS production, resulting in swift bacterial killing (Price et al., 2021). Human neutrophils also form NETs and produce IL-1β in response to *L. pneumophila*, supporting a broader role in immunopathology as well as bacterial control (Koutantou et al., 2025). Interestingly, *L. longbeachae* evades neutrophil microbicidal responses, underscoring species-specific strategies to modulate this early barrier (Hanford et al., 2025). These experimental data place neutrophils alongside alveolar macrophages as central players in shaping early innate immunity and determining the outcome of *L. pneumophila* lung infection.

### 8.4 Comparative host responses in amoebae and human macrophages

Although *L. pneumophila* exploits conserved eukaryotic processes to establish a replication-permissive vacuole, the outcome of infection differs substantially between protozoan hosts and mammalian macrophages (Swanson and Hammer, 2000; Price et al., 2024; Greub and Raoult, 2004; Shames, 2023). Amoebae lack canonical caspase-dependent inflammatory cell death pathways, and *L. pneumophila* replication within protozoan hosts is therefore not associated with pyroptosis or cytokine-driven immune signaling (Gao and Abu Kwaik, 2000b; Shames, 2023; Greub and Raoult, 2004; Gao and Abu Kwaik, 2000a). Instead, bacterial replication within amoebae is primarily constrained by nutrient availability, encystation, and non-inflammatory host stress responses that permit long-term bacterial persistence without host cell lysis (Kilvington and Price, 1990; Boamah et al., 2017). In contrast, infection of mammalian macrophages triggers layered innate immune pathways, including caspase-1-dependent inflammasome activation, species-specific noncanonical inflammasome signaling, NF-κB-driven transcriptional programs, and perturbations of host cell cycle and metabolic state, many of which restrict bacterial replication while simultaneously contribute to inflammatory pathology (Shames, 2023; Akhter et al., 2009; Case et al., 2013; Ge et al., 2009; Bass et al., 2023). Importantly, several of these mammalian responses may or may not have evolved from dedicated adaptation of *L. pneumophila* to vertebrate immunity, but from the translocation of amoeba-adapted effector activities within an evolutionarily divergent host context. This possibility underscores how protozoan selection has shaped bacterial virulence mechanisms that manifest as either permissive or restrictive outcomes depending on host immune architecture (Swanson and Hammer, 2000; Price et al., 2024, Price et al., 2020; Shames, 2023).

## 9 Conclusions

**Figure 2:**
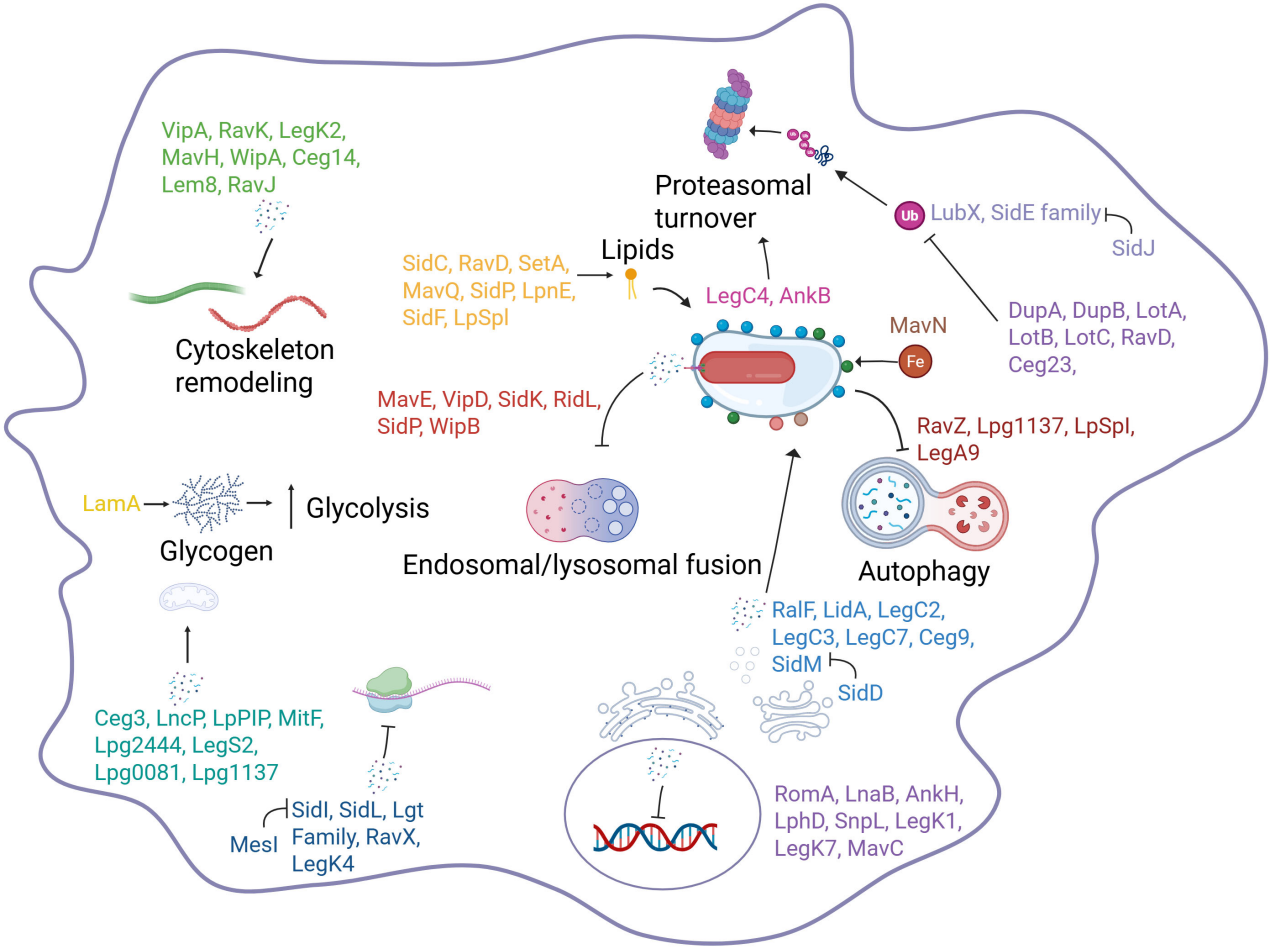
*L. pneumophila* effector targets in macrophage. Following translocation via the Dot/Icm Type IV Secretion System (T4SS), *L. pneumophila* injects over 350 effectors that remodel the host cell environment to promote LCV biogenesis, immune evasion, and nutrient acquisition. Effectors modulate diverse host processes including membrane trafficking and ER recruitment, inhibition of endosomal-lysosomal degradation pathway, and cytoskeleton remodeling. Despite the vast amount of research characterizing the effectors that are shown here, these only represent about 20% of all effectors.

*L. pneumophila* is an environmentally trained intracellular predator whose virulence traits emerged through sustained interactions and long-term co-evolution with protozoa (Mraz and Weir, 2022; Romanov and O’connor, 2024; Price et al., 2024). This ecological perspective explains the organism’s sophisticated intracellular lifecycle, biphasic differentiation, metabolic plasticity, and vast secretory capacity, all of which are pre-optimized for survival in unicellular phagocytic amoebae hosts (Mraz and Weir, 2022; Romanov and O’connor, 2024; Boamah et al., 2017). At the core of its intracellular program is the Dot/Icm T4SS, which translocates ~350 effectors to orchestrate host vesicle trafficking, translation, autophagy, phosphoinositide signaling, ER remodeling, and ubiquitin-mediated control of immunity (Du Toit, 2019; Qiu and Luo, 2017b; Burstein et al., 2016) (**Figure 2**). The co-existence of para-effectors targeting the same host pathway and metaeffectors imposing temporal control underscores that robust intracellular survival has been the primary selective pressure. Although most effectors have little impact in macrophages when deleted individually, this dispensability is precisely what one would expect from a system honed to function across diverse amoebae hosts. in humans, these amoeba-adapted repertoires collide with innate immune networks that *L. pneumophila* never evolved to counter, shaping both protection and immunopathology during infection. Complementing its intracellular arsenal, the T2SS mediates extracellular proteolysis, acquisition of host nutrients, transfer to secondary hosts, protozoan exit, and non-phagosomal effector export, reinforcing the concept that *Legionella* pathogenesis spans both intracellular and extracellular phases (White and Cianciotto, 2019; Debroy et al., 2006a; Debroy et al., 2006b). The coordination of these secretion systems reveals that virulence is not restricted to vacuolar replication, but includes environmental survival, transmission, and community-level persistence.

Host exploitation by *L. pneumophila* also operates strongly at the metabolic level. The LCV has emerged as a dynamically remodeled nutrient acquisition hub and vesicle traffic that is regulated by both bacterial secretion programs and host metabolic rewiring (Wang et al., 2024; Eisenreich and Heuner, 2016; Price et al., 2014a). Amino-acid auxotrophy, iron acquisition, carbon redistribution, and PHB storage are tightly coupled to its virulence lifecycle, positioning nutritional virulence as a co-equal pillar to effector biology (Wang et al., 2024; Eisenreich and Heuner, 2016). In human infection, this metabolic flexibility may contributes to prolonged intracellular survival, persistence within alveolar macrophages, and the capacity to withstand nurtient limitation imposed by the immune response. Future work that couples effectors genetics with spatially resolved metabolomics and flux analyses will be essential to define which metabolic circuits represent true vulnerabilities versus environmentally entrenched necessities.

In amoebae hosts, *L. pneumophila* exploits highly efficient phagocytic machinery and establishes a replication-permissive vacuole within a cellular environment that lacks inflammasome, caspase-dependent cell death pathways, cytokine signaling, and other defining features of mammalian innate immunity (Hagele et al., 2000; Swanson and Hammer, 2000; Boamah et al., 2017). However, studies in mammalian macrophages indicate that *L. pneumophila* is detected and constrained by mammalian innate immune pathways. Murine studies reveal strong inflammasome-mediated restriction through NAIP5/NLRC4, caspases-1/7, and caspase-11-dependent non-canonical pathways, whereas human macrophages rely on distinct PRR combinations, IRF-driven responses, GBP-mediated vacuolar disruption, and cytokine circuits that differ fundamentally from those in mice (Akhter et al., 2009; Case et al., 2013; Bass et al., 2023). Although human macrophages initially support *L. pneumophila* replication, subsequent selective translation blockade, selective cytokine upregulation, metabolic reprogramming, and IL-1R-dependent bystander activation reveal how amoeba-adapted virulence mechanisms trigger vertebrate innate immune defenses to generate a highly layered host response.

In addition, many Dot/Icm effectors directly modulate NF-κB signaling in mammalian cells while the NF-κB pathways do not exist in amoebae (Ge et al., 2009; Losick et al., 2010). Effectors such as LegK1 and LnaB activate NF-κB signaling while MavC and RavD inhibit NF-κB signaling pathways (Ge et al., 2009; Losick et al., 2010; Gan et al., 2019a; Wan et al., 2019). A possible interpretation may be that these proteins originally evolved to manipulate amoebal cellular pathways that share architectural motifs with mammalian NF-κB networks (Price et al., 2020). In macrophages, these same activities intersect with inflammatory transcription and cell survival programs, producing outcomes that are sometimes beneficial for the bacterium or sometimes strongly host-protective. Thus, modulation of various mammalian-specific processes, which do not exist in unicellular amoebae, by *Legionella* may be an accidental host response to amoebae-adapted effectors, such as the LamA effector that interferes with amoebae encystation but once injected into human macrophages, it triggers a paradoxical pro-inflammatory response. However, it is likely that some of the effectors that target mammalian-specific processes have evolved through interaction of *Legionella* with multi-cellular eukaryotic hosts in the environment prior to adaptation to mammalian hosts. Identifying the amoeba host targets of the effectors will uncover the two possibilities and shed further light on evolution of *Legionella* to infect human macrophages.

Moving forward, the field must pivot toward systems-resolution infection biology, integrating single-cell infection heterogeneity, spatial proteomics at the LCV, time-resolved hierarchical effector secretion mapping, and isotope-traced metabolomics across both protozan and mammalian hosts. Notably, domains from Dot/Icm effectors such as SidC, SidK, AnkX, and others have already been adapted as fluorescent biosensors and protein labeling systems, highlighting the experimental tractability through this approach (Luo et al., 2015; Maxson et al., 2022; Heller et al., 2015). Dissecting virulence at this multi-layered resolution will redefine how other amoebae hosts interpret effector essentiality, reveal host-specific vulnerabilities, and expose metabolic bottlenecks that sustain the replicative niche. *L. pneumophila* pathogenesis represents an emergent property of section-driven cellular remodeling, metabolism-driven niche adaptation, and ecology-driven evolutionary selection. Understanding the organism at these intersections, rather than through isolated pathways, will accelerate translational strategies in therapeutic targeting, water-system risk monitoring, and predictive outbreak prevention. More broadly, *L. pneumophila* serves as a model for understanding how environmental pathogen evolve complex intracellular lifestyles that accidentally translate into human virulence, and how innate immunity responds to a pathogen whose evolution occurred largely outside vertebrate hosts.
